# Small heat shock proteins mediate cell-autonomous and -nonautonomous protection in a *Drosophila* model for environmental-stress-induced degeneration

**DOI:** 10.1242/dmm.026385

**Published:** 2016-09-01

**Authors:** Fumiko Kawasaki, Noelle L. Koonce, Linda Guo, Shahroz Fatima, Catherine Qiu, Mackenzie T. Moon, Yunzhen Zheng, Richard W. Ordway

**Affiliations:** Department of Biology andCenter for Molecular Investigation of Neurological Disorders, The Pennsylvania State University, University Park, PA 16802, USA

**Keywords:** HSP23, Neurodegeneration, Flight, Microtubule, Proteostasis, Glia

## Abstract

Cell and tissue degeneration, and the development of degenerative diseases, are influenced by genetic and environmental factors that affect protein misfolding and proteotoxicity. To better understand the role of the environment in degeneration, we developed a genetic model for heat shock (HS)-stress-induced degeneration in *Drosophila*. This model exhibits a unique combination of features that enhance genetic analysis of degeneration and protection mechanisms involving environmental stress. These include cell-type-specific failure of proteostasis and degeneration in response to global stress, cell-nonautonomous interactions within a simple and accessible network of susceptible cell types, and precise temporal control over the induction of degeneration. In wild-type flies, HS stress causes selective loss of the flight ability and degeneration of three susceptible cell types comprising the flight motor: muscle, motor neurons and associated glia. Other motor behaviors persist and, accordingly, the corresponding cell types controlling leg motor function are resistant to degeneration. Flight motor degeneration was preceded by a failure of muscle proteostasis characterized by diffuse ubiquitinated protein aggregates. Moreover, muscle-specific overexpression of a small heat shock protein (HSP), HSP23, promoted proteostasis and protected muscle from HS stress. Notably, neurons and glia were protected as well, indicating that a small HSP can mediate cell-nonautonomous protection. Cell-autonomous protection of muscle was characterized by a distinct distribution of ubiquitinated proteins, including perinuclear localization and clearance of protein aggregates associated with the perinuclear microtubule network. This network was severely disrupted in wild-type preparations prior to degeneration, suggesting that it serves an important role in muscle proteostasis and protection. Finally, studies of resistant leg muscles revealed that they sustain proteostasis and the microtubule cytoskeleton after HS stress. These findings establish a model for genetic analysis of degeneration and protection mechanisms involving contributions of environmental factors, and advance our understanding of the protective functions and therapeutic potential of small HSPs.

## Abstract (precis)

**Summary:** A *Drosophila* model for environmental-stress-induced degeneration exhibits key features for genetic analysis of degenerative disease mechanisms and reveals new forms of protection mediated by small heat shock proteins.

## INTRODUCTION

Failure of proteostasis is implicated in a wide range of degenerative diseases. As a protection mechanism against misfolded proteins in the cytoplasm, their presence triggers a HS response that includes expression of molecular chaperones to suppress proteotoxicity (Morimoto, 2012; Richter et al., 2010). Insufficient protection may contribute to degenerative disorders such as Parkinson's and Alzheimer's disease, hallmarks of which include failure of cellular proteostasis (Hipp et al., 2014; Labbadia and Morimoto, 2015a), cell-type susceptibility (Saxena and Caroni, 2011; Surmeier et al., 2012), dependence on environmental factors (Burbulla and Krüger, 2011; Cannon and Greenamyre, 2011; Goldman, 2014; Tanner et al., 2014) and onset with aging (Labbadia and Morimoto, 2015a; Taylor and Dillin, 2011). Finally, emerging evidence indicates that cell-nonautonomous mechanisms involving intercellular signaling may promote degeneration and contribute to disease spreading (Brettschneider et al., 2015) or be protective (van Oosten-Hawle and Morimoto, 2014). Despite substantial progress, major gaps remain in our understanding of degenerative and protective mechanisms with regard to cellular proteostasis, cell-type-susceptibility and cell-nonautonomous signaling, as well as the contributions of aging and environmental stress.

Of particular relevance to the present study are mechanisms of proteostasis involving clearance of misfolded and aggregated proteins (Vilchez et al., 2014). These proteins are often modified by ubiquitination, which can target them for degradation. Misfolded proteins can be degraded by the proteasome; however, degradation of aggregates appears to be mediated primarily by autophagy (Lim and Yue, 2015). Through autophagy, cytoplasmic protein aggregates, as well as other cytoplasmic components including organelles, are delivered to the lysosome for degradation (Mizushima et al., 2011). Previous studies have shown that clearance of protein aggregates can be localized to the perinuclear compartment of the cell through microtubule-based transport (Mackeh et al., 2013).

As in the present study, previous work has established important roles for cellular stress response pathways in protection from degeneration. Protection has been achieved by manipulating components of the HS response pathway, including members of each major class of eukaryotic HSP: HSP70 (Bonini, 2002; Warrick et al., 1999) and HSP90 (van Oosten-Hawle and Morimoto, 2014), as well as small HSPs (Hagemann et al., 2009; Kourtis et al., 2012; Wang et al., 2011). In the case of HSP90 and HSP70, protection has been shown to include cell-nonautonomous mechanisms (Bobkova et al., 2015; Takeuchi et al., 2015; van Oosten-Hawle et al., 2013). Because of potential therapeutic applications of HSPs (Kakkar et al., 2014; Lindberg et al., 2015; Pratt et al., 2015) and the discovery that mutations in molecular chaperone genes can be causative in degenerative disease (Blumen et al., 2012; Evgrafov et al., 2004; Kakkar et al., 2014), understanding the protective mechanisms mediated by these proteins may contribute to the development of targeted therapies.

The present work employs a new genetic model in which environmental stress in the form of HS induces loss of flight ability through selective degeneration of neurons, glia and muscle cells comprising the *Drosophila* flight motor. This model is attractive for analysis of key problems in the study of degenerative diseases, including cellular proteostasis and proteotoxicity, cell-type susceptibility, cell-autonomous and -nonautonomous protection, and sensitivity to environmental stress. Our studies have already revealed a unique organismal response to an environmental stress, as well as new forms of protection mediated by small heat shock proteins. Future work will further define the mechanisms of HS-stress-induced degeneration and protection, as well as their relationships to genetic factors in degeneration and their relevance to degenerative disease.

## RESULTS

**Fig. 1.**
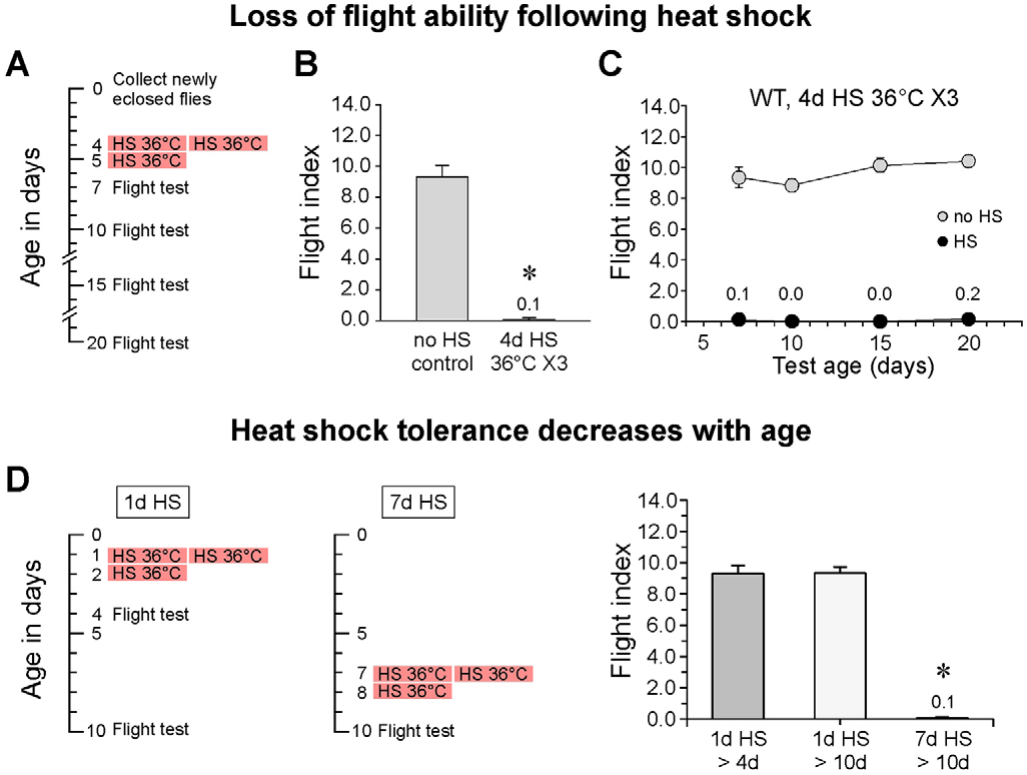
**Loss of flight ability following heat shock.** (A) A standard HS and testing paradigm for HS of 4-day-old flies (4d HS). Groups of six flies were exposed to a series of three 2-hour HS events at 36°C (X3). These occurred at 10:00 a.m. and 2:00 p.m. on the first day and at 10:00 a.m. on the second day. A single flight test was performed at one of the ages indicated. (B) Loss of flight ability in 7-day-old flies after HS at 4 days old. (C) Long-term performance after HS at 4 days was examined according to the paradigm shown in A. WT, wild type. (D) HS tolerance decreases with age. Left, standard HS paradigms for 1-day- or 7-day-old flies. Right, loss of flight ability was not observed in 1-day-old flies exposed to a standard HS paradigm (1 day HS) but did occur in flies subjected to heat shock at 7 days old. In this and the remaining figures, error bars indicate s.e.m. In panels B and D, asterisks denote a significant difference (*n*=10; *P*≤0.01; Student's *t*-test) from the corresponding no-HS control value. In panel C, HS values were significantly different from the no-HS values at every test age (*n=*10; *P*≤0.01; Student's *t*-test). Data are mean±s.e.m.

During the course of our studies in *Drosophila*, we noticed that wild-type control flies exposed to a series of three HS events (hereafter referred to as HS flies) lost flight ability (Fig. 1A,B). Although these HS flies were permanently flightless (Fig. 1C), they retained a high level of basic motor function, as confirmed by general observations and climbing assays (Movie 1 and Fig. S1), and remained fertile. This chance observation raised interesting questions about how a global environmental stress can produce a selective loss of flight ability. It also intersected with our long-standing interest in the cellular and molecular basis of synaptic function at adult neuromuscular synapses of the flight motor (see Kawasaki et al., 2011; Kawasaki and Ordway, 2009). The loss of flight ability following HS stress was dependent on age (Fig. 1D). Comparison of flies exposed to a standard HS stress paradigm starting at either 1 or 7 days old and tested for their flight ability two days after the third exposure to HS [subjected to heat shock at 1 day old and analyzed at 4 days old (1d HS>4d) or subjected to heat shock at 7 days old and analyzed at 10 days old (7d HS>10d), respectively] demonstrated that young flies were strongly resistant to HS stress and retained the flight ability. In contrast, complete loss of flight ability was observed in the older flies. These findings extend those of previous studies reporting loss of flight ability under HS stress (Krebs and Thompson, 2006) and formed the basis for analysis of the underlying cellular and molecular mechanisms.

### Why can't they fly?

**Fig. 2.**
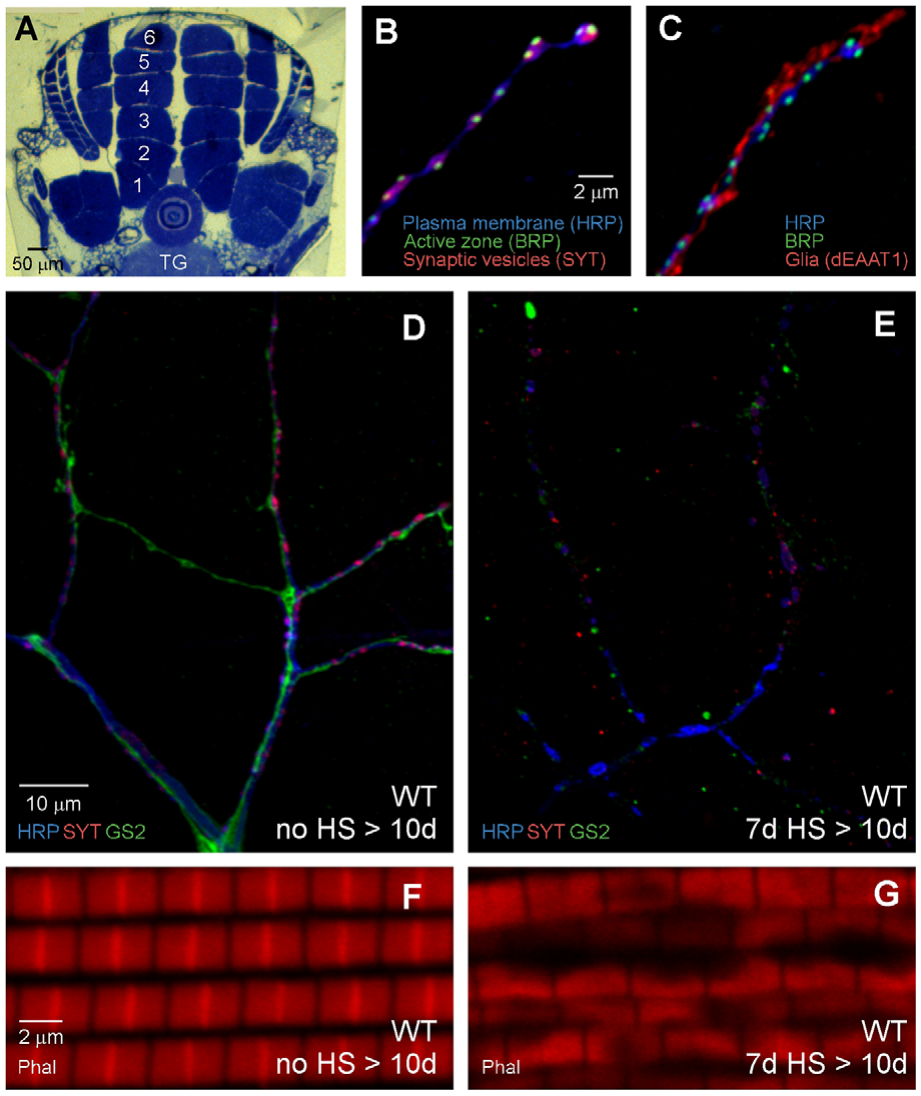
**HS stress induces degeneration of motor axons, glia and muscle fibers in the flight motor.** (A-D,F) Neuronal, glial and muscle cells comprising tripartite DLM neuromuscular synapses. (A) Morphology of the thorax showing two stacks of six DLM fibers in a cross section. These muscles run longitudinally within the dorsal thorax on each side of the midline. Motor axons project from the thoracic ganglion (TG) of the CNS to the DLM muscle fibers (numbered 1-6). (B,C) Axon terminals. Confocal immunofluorescence images of DLM neuromuscular synapses using markers for the neuronal plasma membrane (HRP), active zones (BRP), synaptic vesicles (SYT) and glia (dEAAT1). (D) Lower magnification confocal immunofluorescence image of a no-HS control fly at 10 days old (no HS>10d). The image shows both larger nerve and axon branches (lacking synapses) and synaptic terminal branches (HRP, blue; SYT, red), as well as associated glial processes (GS2, green). (F) Confocal fluorescence image from a no-HS fly showing the DLM contractile apparatus stained with phalloidin (Phal). (E,G) Confocal immunofluorescence images showing cell degeneration in the flight motor of flies exposed to the day 7 HS paradigm and processed for immunocytochemistry at 10 days (7d HS>10d). Note the severe degeneration of axons, glia and muscle in comparison to the no-HS control.

**Fig. 3.**
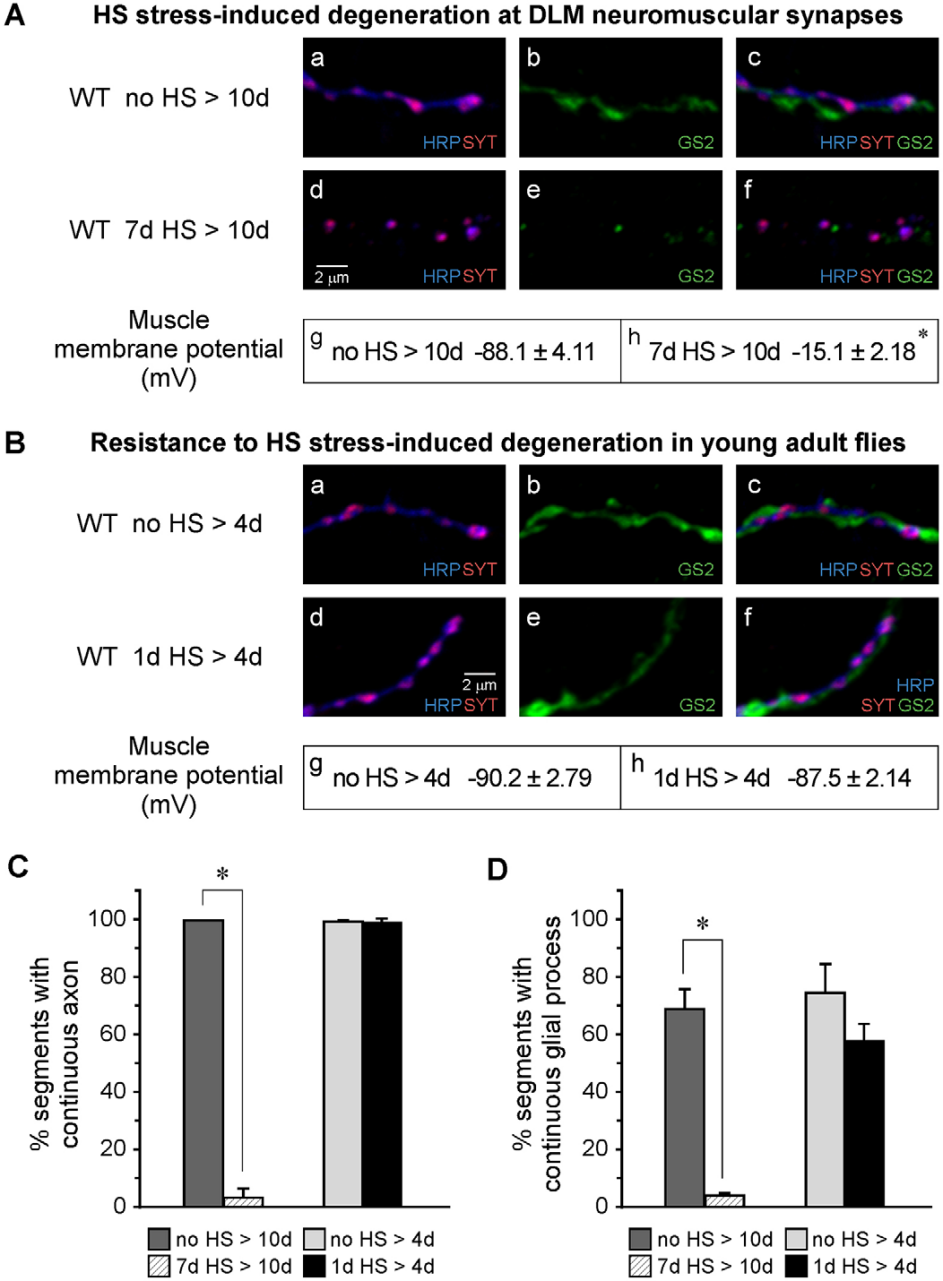
**HS-stress-induced degeneration in the flight motor is age dependent.** (A,C,D) Degeneration in 7-day-old flies. (A) Confocal immunofluorescence images of DLM neuromuscular synapses from ‘no HS>10d’ control flies (Aa-Ac) or ‘7d HS>10d’ flies (Ad-Af) indicating HS-stress-induced degeneration of axonal and glial processes. Recordings of muscle membrane potential (Ag,Ah) indicate loss of muscle viability in ‘7d HS>10d’ flies, consistent with phalloidin staining shown in Fig. 2. (B-D) Young (1-day-old) flies are resistant to HS-stress-induced degeneration. (B) Confocal immunofluorescence images of DLM neuromuscular synapses from ‘no HS>4d' control flies (Ba-Bc) or ‘1d HS>4d’ flies (Bd-Bf), indicating resistance to HS-stress-induced degeneration. Recordings of muscle membrane potential (Bg,Bh) indicate persistence of muscle viability in ‘1d HS>4d’ flies. (C,D) Quantitative comparison of axonal (C) and glial (D) degeneration in ‘7d HS>10d’ or ‘1d HS>4d’ flies with those of the corresponding no-HS controls. Note that only in flies subjected to HS at 7 days, both axonal and glial processes exhibited severe degeneration, indicated by a high degree of fragmentation. In all panels, asterisks indicate a significant difference (*P*≤0.01; Student's *t*-test) from the no-HS control value. The number of experiments was as follows: Ag,Ah (*n*=10); Bg (*n*=8); Bh (*n*=10); C,D (*n*=30). Neuronal (HRP), synaptic vesicle (SYT) and glial (GS2) markers are as in Fig. 2. Data are mean±s.e.m.

Studies of the mechanisms responsible for HS-stress-induced loss of flight ability revealed severe cell degeneration within the flight motor. This work focused on the dorsal longitudinal flight muscle (DLM) as well as DLM motor axons and associated peripheral perisynaptic glia (Fig. 2A-D). These three cell types form DLM neuromuscular synapses, which serve as a model for genetic analysis of molecular mechanisms determining conserved functional properties of synaptic transmission (see Danjo et al., 2011; Kawasaki et al., 2011; Kawasaki and Ordway, 2009; Strauss et al., 2015). For studies of DLM neuromuscular synapse morphology, flies were exposed to a standard HS paradigm starting when they were 7 days old and processed for immunocytochemistry at 10 days old (7d HS>10d). The condition of each cell type was assessed and compared with controls that had not been exposed to HS (no HS>10d) (Fig. 2D-G). In no-HS controls, DLM motor axons, including terminal branches that form neuromuscular synapses, were intact (Fig. 2B,D). Synaptic regions of the axon also exhibited characteristic contacts with perisynaptic glial processes (Fig. 2C,D), as described previously (Danjo et al., 2011; Strauss et al., 2015). With regard to muscle, the apparently healthy condition of DLM fibers in no-HS controls was confirmed through visualization of the contractile apparatus by phalloidin staining (Fig. 2F) or current clamp recordings of DLM membrane potential (see Fig. 3). In contrast to no-HS controls, flies exposed to HS stress exhibited severe degeneration of motor axons, glia and DLM muscle fibers. Both axons and glia were highly fragmented (Fig. 2E), and the muscle contractile apparatus was severely disrupted (Fig. 2G). Degeneration was not evident immediately after the third HS (7d HS>8d) (Fig. S2) but rather developed in parallel for all three cell types over the following two-day period. The observed HS-stress-induced loss of flight ability is not surprising considering the loss of these key cell types in the flight motor. Although HS stress induces loss of flight ability and degeneration within the flight motor, HS flies retained other motor functions and, thus, it was apparent that cell degeneration was not widespread within the organism. Accordingly, examination of the analogous cell types in the leg, including the motor axons, glia and muscle fibers forming a leg neuromuscular synapse, revealed that they are resistant to degeneration for an extended period after HS stress (Fig. S3). Thus neuronal, glial and muscle cell types within the flight motor exhibit selective susceptibility to HS-stress-induced degeneration.

Finally, as observed for loss of flight ability, HS-stress-induced degeneration in the flight motor did not occur in early adulthood. In contrast to 7-day-old flies, those exposed to a standard HS paradigm starting at 1 day old and processed at 4 days old (1d HS>4d) were protected from degeneration. Confocal immunofluorescence images of DLM neuromuscular synapses and recordings of DLM fiber membrane potential (Fig. 3A,B), as well as quantitative comparisons of fragmentation (Fig. 3C,D), showed that old and young flies exhibited striking differences in susceptibility to HS-stress-induced degeneration. Protection in young flies did not result from regeneration, as indicated by the presence of normal cell morphology soon after the third HS (Fig. S4). Thus, HS stress in older flies produces selective degeneration of neurons, glia and muscle cells in the flight motor, leading to selective loss of flight ability.

Several additional features of HS-stress-induced degeneration in the flight motor are noteworthy (Fig. S5). First, degeneration does not depend on flight activity on the basis that wild-type flies do not typically fly during exposure to HS and that HS-stress-induced degeneration has been observed in paralytic flies (Suzuki et al., 1971), which exhibit immediate, complete and sustained paralysis during standard HS treatments (Fig. S5A). Second, neuronal degeneration occurs primarily in the axon on the basis that the motor neuron cell body remains morphologically normal two days after the third HS (Fig. S5B), despite severe fragmentation of terminal axon branches. This is in contrast to degeneration of peripheral perisynaptic glia in which both the cell body and glial processes are peripheral (Strauss et al., 2015) and fully degenerated. Finally, axon degeneration does not appear to occur through Wallerian degeneration (Fig. S5C), a process which is induced following axotomy (Fang and Bonini, 2012; Freeman, 2014).

### HS-stress-induced failure of muscle proteostasis

**Fig. 4.**
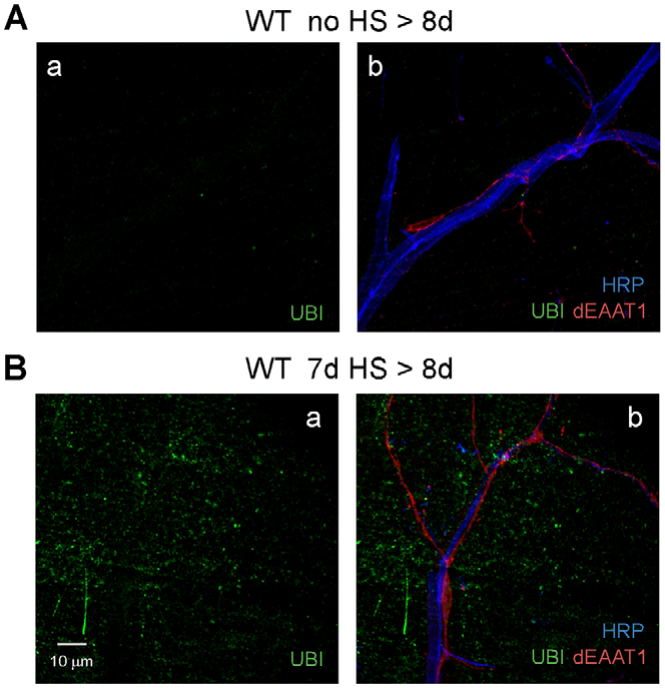
**Failure of muscle proteostasis precedes HS-stress-induced degeneration in the flight motor.** Confocal immunofluorescence images of DLM neuromuscular synapses from ‘no HS>8d’ control flies (A) or ‘7d HS’ flies dissected at 8 days (7d HS>8d) (before degeneration; Fig. S2) (B). Immunostaining for ubiquitin (UBI) indicated that muscle exhibits a marked increase in ubiquitin-positive aggregates after HS stress (Ba) and preceding degeneration (note the presence of intact axonal and glial processes in Bb). Ubiquitin-positive aggregates were not evident in axonal and glial processes. Neuronal (HRP) and glial (dEAAT1) markers are as in Fig. 2.

To examine a possible role for proteotoxicity in HS-stress-induced degeneration, the first step was to determine whether protein aggregation occurs and, if so, whether enhancement of the HS response pathway can suppress degeneration. Wild-type flies at 7 days old were exposed to a standard HS paradigm and processed for immunocytochemistry at 8 days old after the third HS event (7d HS>8d). Exposure to HS stress has been completed at this time point; however, axons, glia and muscle do not yet exhibit morphological signs of degeneration (Fig. S2). In contrast to no-HS controls (Fig. 4A), flies exposed to HS stress exhibited diffuse ubiquitin-positive puncta in DLM fibers (Fig. 4B), indicating widespread aggregation of ubiquitinated proteins. However, leg muscle, which is resistant to HS-stress-induced degeneration, did not exhibit accumulation of ubiquitinated protein aggregates under the same conditions (Fig. S6). Finally, it was of interest to compare failure of proteostasis induced by acute HS stress to that observed in a previous study of aging *Drosophila* flight muscle (Demontis and Perrimon, 2010). In that study, the P62 protein [also known as Ref(2)P] was used as a marker for intermediates in autophagy (Pircs et al., 2012), and an increase in punctate P62 signal in aging muscle indicated an accumulation of intermediates resulting from reduced flux through the pathway. Our studies confirmed that aging DLM fibers exhibit distinct and well-defined ubiquitin-positive puncta that strongly colocalized with P62 (Fig. S7A). In contrast, HS stress produces a diffuse distribution of smaller and less-uniform ubiquitin-positive puncta, which exhibited little colocalization with P62 (Fig. S7B). These findings indicate different effects of aging and acute stress on DLM fiber proteostasis and provide a model in which to examine the interactions of these factors in degeneration.

### Protection from HS-stress-induced degeneration by enhancement of the HS response pathway in muscle or by muscle overexpression of a small heat shock protein, HSP23

**Fig. 5.**
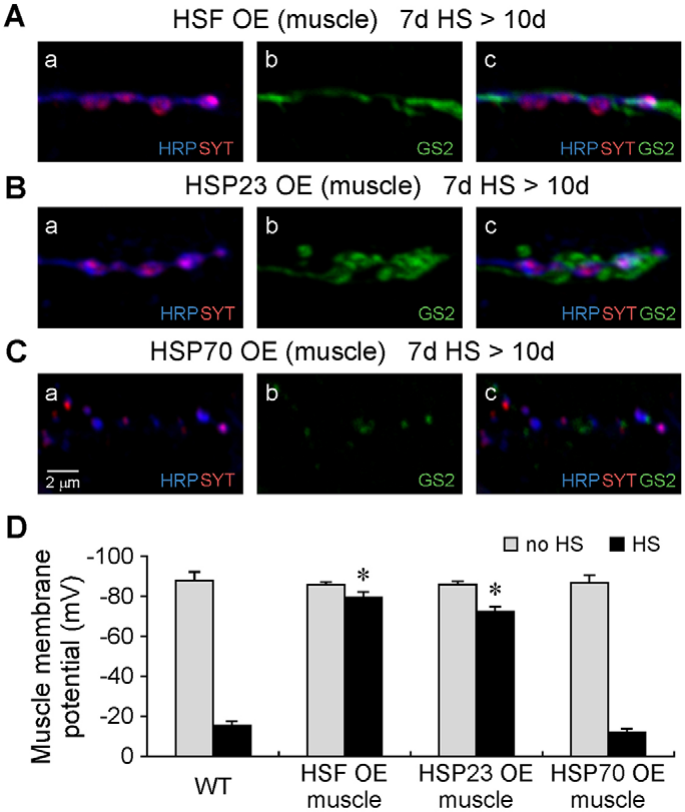
**Protection from HS-stress-induced degeneration by enhancement of the HS response pathway in muscle or muscle overexpression of the small heat shock protein HSP23.** Confocal immunofluorescence images (A-C) and muscle membrane potential recordings (D) from DLM neuromuscular synapses in flies exhibiting muscle overexpression of HSF (HSF OE), HSP23 (HSP23 OE) or HSP70 (HSP70 OE). In HSF OE (muscle) (A,D) or HSP23 OE (muscle) (B,D) flies, cell-autonomous protection of muscle as well as cell-nonautonomous protection of neuronal and glial processes were observed after exposure to a standard HS paradigm (7d HS>10d). In contrast, HSP70 OE (muscle) flies did not exhibit protection (C,D). In D, asterisks indicate a significant difference (*P*≤0.01; Student's *t*-test) from the wild-type (WT) control value. For comparison, the WT control data shown numerically in Fig. 3Ag,Ah were included in the graph. The number of experiments follows: WT [no HS (*n*=10), HS (*n*=10)]; HSF OE [no HS (*n*=6), HS (*n*=6)]; HSP23 OE [no HS (*n*=6), HS (*n*=8)]; HSP70 OE [no HS (*n*=7), HS (*n*=8)]. Neuronal (HRP), synaptic vesicle (SYT) and glial (GS2) markers are as in Fig. 2. Data are mean±s.e.m.

HS-stress-induced failure of proteostasis in flight muscle indicates that enhancement of the HS response pathway might sustain muscle proteostasis and suppress degeneration. Accordingly, muscle-specific overexpression of Heat shock transcription factor (HSF) (Anckar and Sistonen, 2011) was performed using the GAL4-UAS transgenic expression system (Brand and Perrimon, 1993). Muscle-specific overexpression of HSF protected the flight motor from degeneration (Fig. 5). Notably, this included both cell-autonomous protection of muscle and cell-nonautonomous protection of neurons and glia (Fig. 5A,D). To investigate the mechanism of protection mediated by overexpression of HSF in muscle, induction of HSF target genes by muscle-specific overexpression of HSF was assessed by western blotting for two representative molecular chaperones, HSP70 (Gong and Golic, 2006; Solomon et al., 1991) and HSP23 (Garrido et al., 2012; Michaud and Tanguay, 2003) (Fig. S8). Flies overexpressing HSF in muscle [HSF OE (muscle)] exhibited a marked increase in basal HSP23 expression, and this level increased further after exposure to HS. On this basis, we examined whether increased HSP23 expression in muscle is sufficient for protection. Flies overexpressing HSP23 in muscle [HSP23 OE (muscle)] were protected against HS-stress-induced degeneration of muscle, as well as of neurons and glia (Fig. 5B,D), indicating that overexpression of HSP23 in muscle is sufficient for cell-autonomous and -nonautonomous protection. In contrast, HSP70 overexpression in muscle did not protect any of the three cell types (Fig. 5C,D).

Protection of neurons and glia by overexpression of HSF or HSP23 in muscle does not reflect simply a passive effect of muscle viability on the basis of two observations. First, axonal and glial degeneration was not observed after mechanically killing the DLM muscle fibers in 8-day-old flies and processing them for immunocytochemistry at 10 days old (8d stab>10d) (Fig. S9A). Second, DLM neuromuscular synapses in a *parkin* mutant, which exhibits severe degeneration of DLM fibers (Greene et al., 2003), retained intact axonal and glial processes (Fig. S9B). The preceding findings indicate that degeneration of axons and glia requires HS stress, rather than simply muscle degeneration, and that protection can be conferred on these cell types by altering muscle gene expression and protecting DLM fibers. Within the scope of the current study, further analysis focused primarily on cell-autonomous protection of muscles mediated by overexpression of HSP23.

### HSP23 overexpression in muscle promotes proteostasis mechanisms after HS stress

**Fig. 6.**
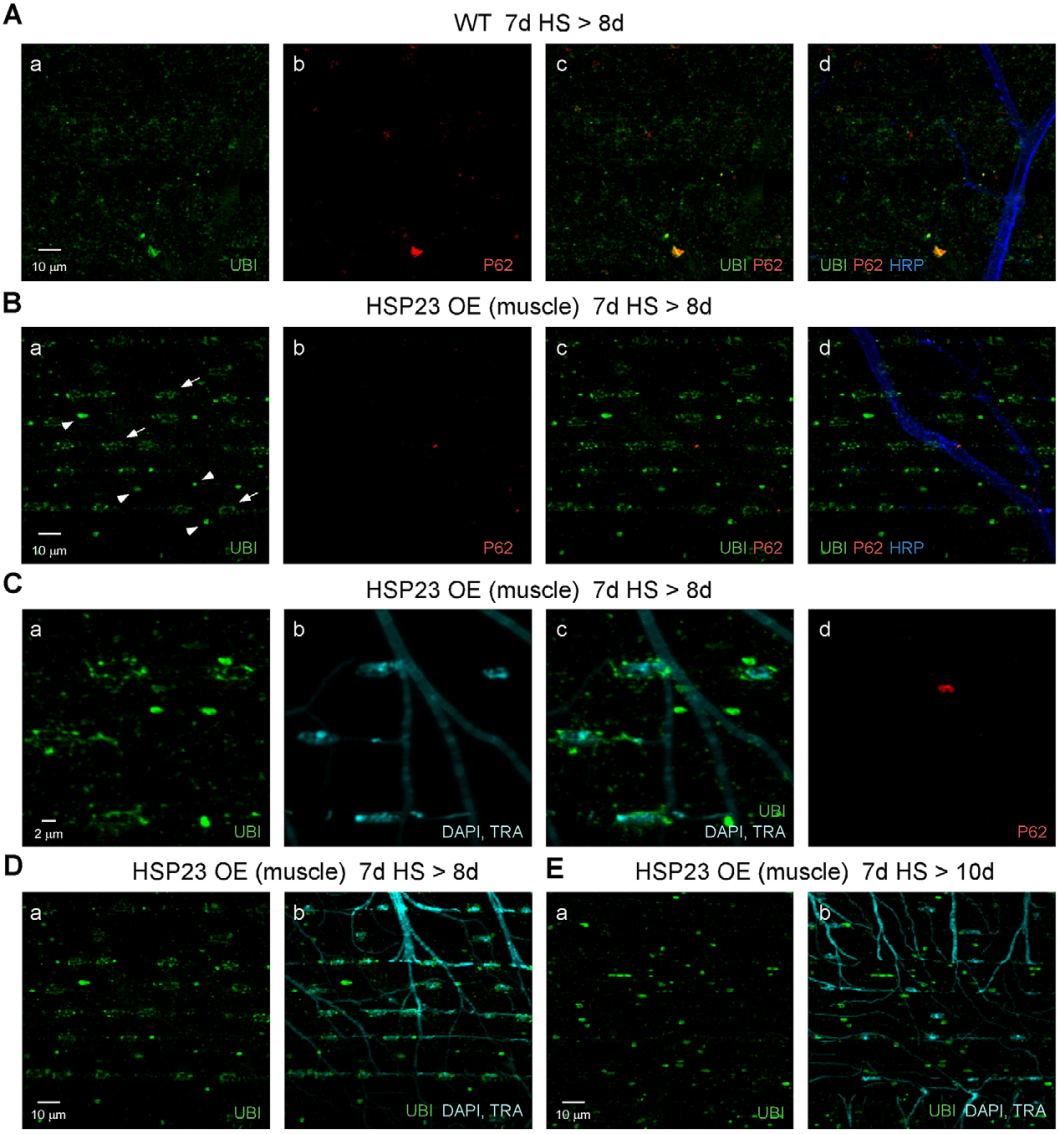
**Overexpression of HSP23 in muscle promotes perinuclear proteostasis mechanisms after HS stress.** Confocal immunofluorescence images of DLM neuromuscular preparations from wild-type (WT) flies (A) or those overexpressing HSP23 in muscle (HSP23 OE; B-E). P62 labels intermediates in autophagy. DAPI labeling of nuclei and autofluorescence from trachea (TRA) appear in the same channel. Ubiquitin (UBI) and neuronal (HRP) markers are as described in Fig. 2. (A-D) flies were exposed to a standard HS stress paradigm and processed before degeneration was observed in WT (7d HS>8d). In contrast to the diffuse distribution of ubiquitinated protein aggregates in WT muscle (A), HSP23 OE flies exhibited well-defined and spatially organized ubiquitin-positive puncta, including ring-like patterns (arrows in Ba) and a distinct class of larger puncta (arrowheads in Ba). Little colocalization of ubiquitin and P62 was observed for either genotype (Ac and Bc). (C) DAPI staining revealed perinuclear rings of ubiquitinated proteins, which are clearly distinguishable from a larger class of non-perinuclear puncta. (D,E) Comparison of ubiquitin-positive puncta in HSP23 OE (muscle) flies early after HS stress (D; 7d HS>8d) and later (E; 7d HS>10d) indicates that perinuclear puncta were cleared whereas non-perinuclear puncta persist.

We considered that HSP23 overexpression might protect muscle by altering proteostasis to redistribute or degrade ubiquitinated protein aggregates induced by HS stress. This possibility was addressed by examining the distribution of ubiquitinated protein aggregates and their spatial relationship to the P62 marker for intermediates in autophagy (Fig. 6). Wild-type and HSP23 OE (muscle) flies were exposed to a standard HS paradigm at 7 days old and processed for immunocytochemistry after the third HS (7d HS>8d). This time point precedes morphological signs of degeneration in unprotected wild-type preparations (Fig. S2). In comparison with wild type (Fig. 6A), HSP23 OE (muscle) flies exhibited a striking distribution of ubiquitinated proteins, including smaller puncta arranged in ring-like configurations (arrows in Fig. 6Ba) and a distinct class of larger puncta (arrowheads in Fig. 6Ba). Both classes exhibited little colocalization with P62 (Fig. 6Bb,Bc). Further examination indicated that the ubiquitin-positive rings represent a perinuclear distribution (Fig. 6Ca,Cc) and thus defined distinct classes of perinuclear and non-perinuclear puncta. Finally, HSP23 OE (muscle) flies were examined two days after HS stress (7d HS>10d) to determine the fate of both classes of ubiquitin-positive puncta (Fig. 6D,E). Notably, perinuclear, but not non-perinuclear, puncta had been cleared at this later time point, suggesting a protective role for perinuclear puncta in resolution or degradation of ubiquitinated proteins. The following studies further defined these perinuclear and non-perinuclear ubiquitin-positive puncta by examining their spatial relationships to markers for subcellular compartments in the multinucleate DLM.

The distribution of non-perinuclear ubiquitin-positive puncta was considered relative to the whole muscle fiber morphology. Myofibrils are the predominant feature, and these are surrounded by a densely packed population of mitochondria as well as interspersed nuclei (Fig. S10A). Further analysis using a mitochondrial marker, mito-DsRed, indicated that non-perinuclear puncta represent ubiquitinated mitochondria (Fig. S10B). Thus overexpression HSP23 in muscle promotes increased ubiquitination of mitochondria. Note that the reduced mito-DsRed signal in ubiquitinated mitochondria (insets in Fig. S10Bc,Bd) indicates that protection involves clearance of mitochondria, possibly through a form of autophagy known as mitophagy (Mizushima et al., 2011).

**Fig. 7.**
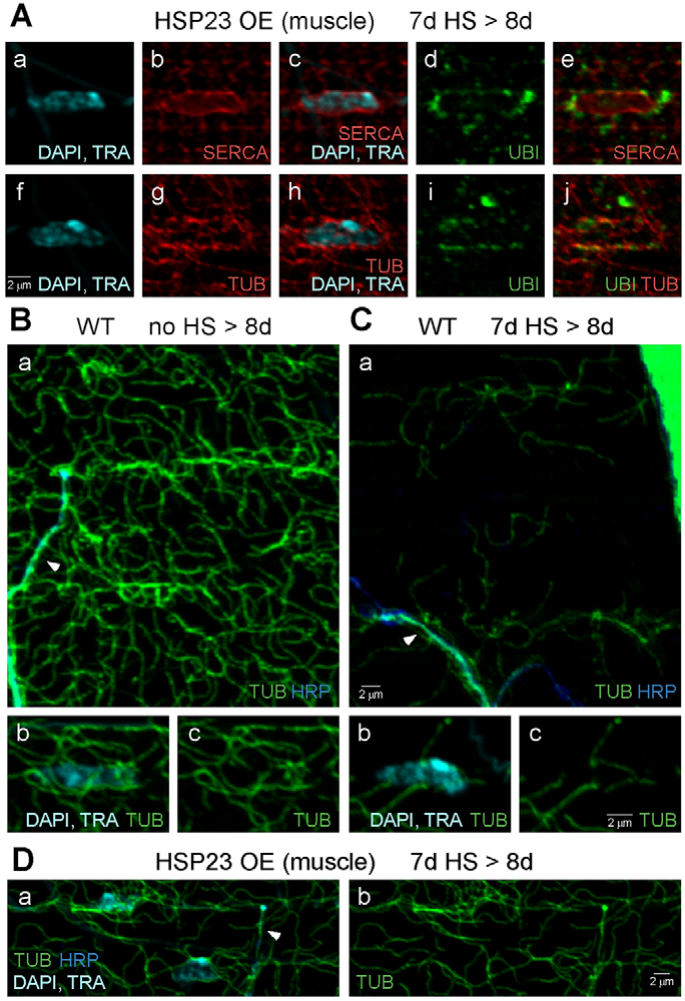
**Perinuclear ubiquitin-positive aggregates in muscle interact with a perinuclear microtubule network that is disrupted by HS stress.** Confocal immunofluorescence images of DLM neuromuscular preparations from flies overexpressing HSP23 in muscle (HSP23 OE) (A,D) or wild-type (WT) flies (B,C). SERCA labels the sarcoplasmic reticulum (SR) and nuclear envelope, and tubulin (TUB) labels microtubules. (A) The distribution of perinuclear ubiquitinated protein aggregates in HSP23 OE (muscle) flies after a standard HS paradigm and processing after the third HS (7d HS>8d). Aggregates were localized to the cytoplasmic surface of the SR (Aa-Ae) and closely associated with the perinuclear microtubule network (Af-Aj). (B) The muscle microtubule cytoskeleton (Ba) and perinuclear microtubule network (Bb,Bc) in WT control flies (no HS>8d). An axon branch containing microtubules is shown (arrowhead). (C) The muscle microtubule cytoskeleton (Ca) and perinuclear microtubule network (Cb,Cc) in WT flies exposed to a standard HS paradigm and processed before degeneration (7d HS>8d). Severe loss of the muscle microtubule cytoskeleton (Ca) and perinuclear microtubule network (Cb,Cc) was observed after HS stress and prior to degeneration. In contrast, the axonal microtubule cytoskeleton (arrowhead) remained present after HS stress. (D) HSP23 OE (muscle) flies exposed to a standard HS stress paradigm at 7 days old and processed after the third HS (7d HS>8d) exhibited protection of the muscle microtubule cytoskeleton from HS stress. Neuronal (HRP), nuclear (DAPI) and tracheal (TRA) markers are as described in previous figures.

The distribution of perinuclear puncta, which may represent cytoplasmic aggregates of ubiquitinated proteins, was determined relative to the sarcoplasmic reticulum (SR), which surrounds nuclei and forms the nuclear envelope (Fig. 7Aa-Ae). They were found to reside outside of the SR but in close proximity to its surface. Because these ubiquitinated protein aggregates can be transported to perinuclear regions, their localization was also examined relative to a microtubule marker (Fig. 7Af-Aj). They were found in close association with a dense network of perinuclear microtubules as well as surrounding microtubule tracks. These results suggest that overexpression of HSP23 in muscle supports proteostasis mechanisms involving microtubule-based transport of ubiquitinated protein aggregates to the perinuclear compartment and their subsequent clearance.

Finally, the possibility of role for microtubule-based transport in perinuclear proteostasis mechanisms suggests that disruption of the muscle microtubule cytoskeleton might contribute to HS-stress-induced failure of proteostasis and degeneration. This possibility was examined in wild-type flies exposed to a standard HS paradigm at 7 days old and processed for immunocytochemistry after the third HS (7d HS>8d). This time point precedes morphological degeneration in the flight motor (Fig. S2). Severe disruption of the muscle microtubule cytoskeleton was observed after HS stress, including loss of the perinuclear microtubule network (Fig. 7B,C). Thus, disruption of the muscle microtubule cytoskeleton is an early HS-stress-induced event which, together with accumulation of diffusely distributed ubiquitinated protein aggregates in muscle (Fig. 4B), precedes morphological degeneration. In contrast to these observations in wild type, the muscle microtubule cytoskeleton in HSP23 OE (muscle) flies was resistant to HS stress (Fig. 7D). These findings suggest HSP23 overexpression in muscle confers protection, in part, by preserving the muscle microtubule cytoskeleton. This mechanism may support perinuclear localization and clearance of ubiquitinated proteins, and prevent the diffuse distribution of ubiquitinated protein aggregates observed in wild type.

### Endogenous protection mechanisms in young adult flies

**Fig. 8.**
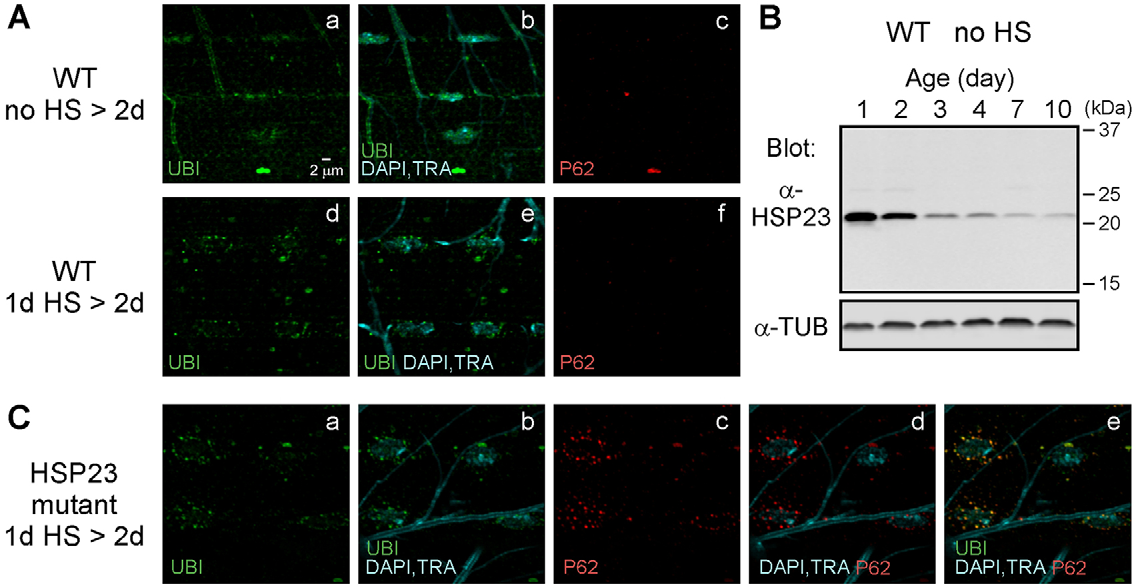
**Endogenous protection mechanisms in young flies.** (A) Proteostasis in young WT flies after HS stress resembles that observed in older flies protected by HSP23 overexpression in muscle. Confocal immunofluorescence images of DLM neuromuscular synapse preparations. In the muscle of control flies (no HS>2d) (Aa-Ac), ubiquitin (UBI) signal was detected within nuclei (Aa,Ab); however, neither perinuclear nor non-perinuclear ubiquitin-positive puncta were observed (Aa,Ab). In contrast, the muscle of flies exposed to a standard HS stress paradigm at 1 day old (1d HS>2d) (Ad-Af) exhibited both perinuclear and non-perinuclear ubiquitin-positive puncta, which resembled those of older flies protected by HSP23 overexpression in muscle. Like those, they were P62-negative. (B) Western blot analysis of endogenous HSP23 expression in homogenates of WT no-HS control flies showed that young adults exhibit higher levels of basal HSP23 expression. Tubulin (TUB) was used as a loading control. (C) Loss of endogenous HSP23 function in young flies alters muscle proteostasis after HS stress. Confocal immunofluorescence images of DLM neuromuscular preparations showing that muscle of young *hsp23*-mutant flies exposed to a standard HS stress paradigm at 1 day old (1d HS>2d) accumulates perinuclear ubiquitin-positive puncta, which are P62-positive intermediates in autophagy. Markers for ubiquitin (UBI), nuclei (DAPI), trachea (TRA) and P62 are as described in previous figures.

As discussed in the preceding results, young wild-type flies are resistant to HS-stress-induced degeneration in the flight motor (Fig. 3). To examine the relationship of this endogenous protection to that of HSP23 overexpression in the muscle of older flies, the subcellular distribution of ubiquitinated proteins was examined in young wild-type flies protected from degeneration. Wild-type flies were exposed to a standard HS stress paradigm at 1 day old and processed for immunocytochemistry after the third HS (1d HS>2d). Endogenous mechanisms of muscle proteostasis, as assessed by the distribution of ubiquitinated proteins, were similar to those observed in older HSP23 OE (muscle) flies. The DLM fibers of young wild-type flies exhibited perinuclear and non-perinuclear ubiquitin-positive puncta (Fig. 8A) that were negative for P62. Moreover, western blotting of homogenates from wild-type flies of different ages demonstrated a high level of basal HSP23 expression in young adults (Fig. 8B). On this basis, we examined whether loss of endogenous HSP23 function in young flies influences endogenous protection. *hsp23*-mutant flies exposed to a standard HS paradigm at 1 day old demonstrated both protection from degeneration and persistence of the muscle microtubule cytoskeleton (Fig. S11), indicating that other endogenous mechanisms in young adult flies are sufficient for protection. However, these experiments revealed a clear difference between wild-type and *hsp23*-mutant flies with respect to the observed perinuclear ubiquitinated protein aggregates (Fig. 8C). In the absence of endogenous HSP23, perinuclear puncta exhibited strong colocalization with P62, indicating that they represent intermediates in clearance of ubiquitinated protein aggregates through autophagy. Taken together, the preceding findings indicate that HSP23 is necessary for efficient clearance of ubiquitinated protein aggregates through a perinuclear mechanism involving autophagy.

## DISCUSSION

Here, we report that environmental stress in the form of HS produces a selective loss of flight ability and selective degeneration of neuronal, glial and muscle cells within the flight motor. Degeneration was preceded by a failure of proteostasis in muscle and disruption of the muscle microtubule cytoskeleton. Muscle-specific overexpression of a small heat shock protein, HSP23, provided cell-autonomous protection of muscle as well as nonautonomous protection of neurons and glia. Cell-autonomous protection of muscle promoted proteostasis mechanisms involving perinuclear localization and clearance of ubiquitinated proteins, persistence of the microtubule cytoskeleton and increased ubiquitination of mitochondria. These findings establish a genetic model for environmental-stress-induced degeneration and open many avenues of exploration. Among these, the primary focus of the present study extends from defining this new experimental model to cell-autonomous protection mediated by HSP23 in muscle.

The roles of HSP23 in muscle proteostasis and cell-nonautonomous protection of neurons and glia provide new insight into the *in vivo* functions of small HSPs. HSP23 overexpression in flight muscle prevented the failure of muscle proteostasis and the microtubule cytoskeleton that precedes degeneration, indicating small HSPs can play important roles in organizing proteostasis mechanisms after HS stress. Consistent with these findings, small HSPs have been shown to promote formation, transport and autophagic degradation of ubiquitinated protein aggregates (Gamerdinger et al., 2011). Similar proteostasis mechanisms, as well as persistence of the microtubule cytoskeleton, were observed endogenously in the protected flight muscle of young wild-type flies (Fig. 8A and Fig. S11B) in which basal levels of endogenous HSP23 were elevated (Fig. 8B). Analysis of young adult *hsp23* mutants indicated that endogenous HSP23 participates in clearance of perinuclear ubiquitinated protein aggregates (Fig. 8C); however, these flies remained resistant to HS-stress-induced degeneration. This may reflect overlapping functions of the twelve *Drosophila* small HSPs (Morrow and Tanguay, 2015) or other protective mechanisms operating in early adulthood. An analogous form of protection in young adults of *Caenorhabditis elegans* is mediated through a cell-nonautonomous protective effect of the gonad on somatic tissue (Labbadia and Morimoto, 2015b). With regard to cell-nonautonomous signaling in the present study, early disruption of muscle proteostasis and microtubule cytoskeleton suggests a primary role for muscle in flight motor degeneration. However, neuronal and glial degeneration are not induced by muscle degeneration alone (Fig. S9), but only in combination with HS stress. Future studies will examine whether HSP23 participates directly in cell-nonautonomous signaling to neurons and glia or indirectly by maintaining muscle viability. Finally, the present findings intersect with those of another study in *C. elegans* (Kourtis et al., 2012). A single severe HS (39°C for 15 min) is lethal to the majority of worms, and survivors exhibit widespread degeneration. Ubiquitous overexpression of either HSF or the small heat shock protein HSP-16.1 increases survival and provides protection from degeneration. It will be interesting to determine whether small-HSP-mediated protection in *C. elegans* involves cell-autonomous proteostasis mechanisms similar to those reported here, whether small HSPs provide cell-nonautonomous protection and whether this model exhibits susceptibility of specific cell types.

Cell-type susceptibility is an interesting feature of the experimental model reported here and an important problem in the study of degenerative disease. One example is selective degeneration of certain dopaminergic neurons in Parkinson's disease, which may reflect specific features of this cell type, including its high metabolic burden and physiological properties (Surmeier et al., 2012). Regarding HS-stress-induced degeneration in the present study, neuronal, glial and muscle cells controlling leg motor function were resistant to HS-stress-induced degeneration (Fig. S3B), and leg muscle did not exhibit either accumulation of ubiquitinated protein aggregates (Fig. S6) or loss of the microtubule cytoskeleton (Fig. S12). These observations suggest that the same global stress produces protein misfolding and aggregation in susceptible flight muscle but not resistant leg muscle, and indicate important differences in either the load of misfolded proteins or the capacity to process them. Note that DLM fibers are exceptionally large cells (Fig. 2A) with a high density of large mitochondria (Fig. S10A). Even apart from the metabolic demands of flight, these cells are expected to have a high metabolic burden and could be at higher risk with regard to failure of energy-dependent processes such as protein refolding and microtubule assembly. Selective degeneration of flight muscles in the *parkin* mutant and several other genetic models of degeneration (Lloyd and Taylor, 2010) (see also Fig. S9B) indicate that these cells are inherently susceptible to both genetic perturbation and environmental stress. Finally, the DLM motor neurons, most of which innervate an entire DLM fiber (Ikeda and Koenig, 1988; Sun and Wyman, 1997), and the peripheral perisynaptic glia (Strauss et al., 2015) branch extensively over the entire muscle surface. The large arbors maintained by these cells may, in a manner analogous to those of dopaminergic neurons, contribute to their susceptibility.

Several features of this new genetic model for environmental-stress-induced degeneration have the potential to advance our understanding of degenerative and protective mechanisms. Powerful genetic approaches in *Drosophila*, including cell-type-specific genetic analysis and forward genetic screens, can be used to address mechanisms of degeneration and protection, cell-type susceptibility and cell-nonautonomous signaling. These studies benefit from the simple and accessible network of susceptible cell types within the flight motor and the corresponding resistant cell types participating in leg motor function. Another important advantage of this model is its ability to induce degeneration with a well-defined and acute environmental stress. This provides an initial reference point in the temporal series of events leading to degeneration, for example the early failures of proteostasis (Fig. 4) and the microtubule cytoskeleton (Fig. 7B,C) in muscle. Thus, temporal changes in the transcriptome and proteome might provide insight into the molecular mechanisms participating in degeneration and their interrelationships. Finally, previous studies in *Drosophila* have provided a wide range of models for degenerative disease (Frost et al., 2015; McGurk et al., 2015), as well as advances in understanding the cellular and molecular basis of aging (Alic et al., 2014; Demontis and Perrimon, 2010). Together with these previous studies, the present work creates new opportunities to examine interactions of environmental stress, genetics and aging in degeneration.

## MATERIALS AND METHODS

### *Drosophila* genetics

The GAL4-UAS system (Brand and Perrimon, 1993) was used for transgenic expression. GAL4 lines included the muscle driver Mhc-GAL4 (Schuster et al., 1996), a neuronal driver including elav-GAL4 (C155) (Lin and Goodman, 1994) and appl-GAL4 (Torroja et al., 1999) recombined onto the same X chromosome and a DLM motor neuron driver, D42-GAL4 Cha-GAL80 (Vonhoff et al., 2013) [Fernando Vonhoff (Yale University, New Haven, CT)]. UAS transgenes included UAS-HSP23, UAS-mCD8-GFP and UAS-mito-GFP [Bloomington *Drosophila* Stock Center (Indiana University, Bloomington, IN)]; UAS-HSP70-myc 9.1 (Xiao et al., 2007) [R. Meldrum Robertson (Queen's University, Kingston, Ontario, Canada)]; UAS-Wlds (Hoopfer et al., 2006) [Liqun Luo (Stanford University, Stanford, CA)]; UAS-mito-DsRed (Lutas et al., 2012) and UAS-HSF-GFP on the X chromosome (UAS-HSF-GFP-R1) generated by mobilizing an existing third chromosome transgene from the Lis lab (Yao et al., 2006) [John Lis (Cornell University, Ithaca, NY)]. Mutant lines included *hsp23* LL01535 (Kyoto *Drosophila* Stock Center, Kyoto, Japan) and *parkin^25^* (Greene et al., 2003) [Leo Pallanck (University of Washington, Seattle, WA)]. These mutations were studied *in trans* to the following deficiencies, respectively: Df(3L) AC1 and Df (3L) BSC733 [Bloomington *Drosophila* Stock Center (Indiana University, Bloomington, IN)]. The *paralytic^TS1^* mutant (Suzuki et al., 1971) is from our laboratory stock collection. Experiments were performed using female flies. Wild-type flies were Canton-S. Stocks and crosses were cultured on a conventional cornmeal-molasses-yeast medium at 20°C in a 12-h day and 12-h night cycle.

In experiments involving the GAL4-UAS system, control experiments were performed in all cases to confirm that phenotypes were not observed in flies carrying only the GAL4 driver or the UAS transgene. The HS-stress-induced degeneration phenotype observed in our laboratory Canton-S stock was confirmed in other wild-type strains, including the following, available from the Bloomington *Drosophila* Stock Center: Canton-S (#1), Canton-S-iso2B (#9514) and Oregon-R-P2 (#2376).

### Heat shock

As indicated in Fig. 1, standard heat shock paradigms involved a series of three 2-h HS events at 36°C and varied only in the age at which the procedure started (1 day, 4 days or 7 days). The first two HS events were started at 10:00 a.m. and 2:00 p.m. on the first day, and the third heat HS was started at 10:00 a.m. on the second day. Newly eclosed flies were collected during a time window between 12:00 p.m. and 3:00 p.m., and stored in standard food vials at 20°C in a 12-h day 12-h night cycle. For HS at 4 days old, flies were transferred to fresh vials on day 3. For HS at 7 days old, flies were transferred to fresh vials on day 5. HS was performed by placing standard food vials plugged with rayon in a circulating water bath preheated to 36°C. Care was taken to ensure the rayon plugs were advanced to below the water level. Following HS, flies were maintained at 20°C in a 12-h day 12-h night cycle. For long-term behavioral tests, flies were transferred to fresh vials every five days.

### Behavior analysis

#### Flight assay

The flight assay was based on previous studies (Atkinson et al., 2000). A simple apparatus was constructed from a cylindrical glass chamber with an open top and internal dimensions of 9-cm diameter and 19-cm height. The height was marked on the exterior surface with horizontal rings at 1-cm intervals starting at a zero line 2 cm above the bottom of the internal chamber. Oil was added to the chamber, and the cylinder was tilted and rotated such that oil coated the walls and filled the bottom to the zero line. The walls were recoated every 5 min. A lid placed over the open top of the cylinder had a hole in the center for introducing flies. After inverting a fly vial over the hole, the vial was tapped or rotated rapidly to move flies into the cylinder. Fliers stuck to the walls of the cylinder at a height related to flight ability, and non-fliers dropped to the bottom. Each test included six flies, and the height of each was determined to the closest integer value. The mean height was recorded for each test and these values were averaged over multiple tests to obtain the flight index (FI). An FI of 10 indicates that flies stuck 10 cm above the zero line on average. As shown, in some cases the FI for HS flies was normalized to that of no-HS controls. FI values represent data from tests on ten independent groups of six flies.

#### Climbing assay

Climbing tests were performed in a 100-ml graduated cylinder (Pyrex) with an inner diameter of 2.5 cm (Corning, Tewksbury, MA). A group of six flies was tapped to the bottom of the cylinder, and the time for half of the flies to climb to the 40 ml mark (∼7.3 cm) was recorded. For each test, this process was repeated three times to produce a mean value. The mean values were averaged over multiple tests, and the inverse was taken to obtain a climbing index (CI) such that larger values represent faster climbing. CI values represent data from tests on ten independent groups of six flies.

### Immunocytochemistry

DLM neuromuscular synapse preparations were examined by immunocytochemistry essentially as described previously (Kawasaki et al., 2004). Imaging was performed using an Olympus FV1000 confocal microscope (Olympus Optical, Tokyo, Japan) with a PlanApo 60×1.4 numerical aperture oil objective (Olympus Optical) and a *z*-step size of 0.2 µm. The same methods were utilized to examine coxal muscle neuromuscular synapses in the leg. Images were obtained and processed with Fluoview software (Olympus Optical). Images shown are representative of those obtained from at least three different preparations.

#### Antibodies and reagents

Rabbit anti-Synaptotagmin Dsyt CL1 (α-SYT; 1:5000; Dr Noreen Reist, Colorado State University, Fort Collins, CO; Mackler et al., 2002); rabbit anti-dEAAT1 (1:2500; Dr Serge Birman, Developmental Biology Institute of Marseille, France; Rival et al., 2006); monoclonal antibody (mAb) nc82 anti-BRP (BRUCHPILOT; 1:50; Developmental Studies Hybridoma Bank, Iowa City, IA; Wagh et al., 2006); mAb GS-6 anti-Glutamine Synthetase (α-GS2) (MAB 302; 1:500; EMD Millipore, Billerica, MA); goat anti-HRP antibody conjugated to Alexa-Fluor-647, which labels neuronal plasma membranes (123-605-021; 1:200; Jackson Immunoresearch Laboratories, West Grove, PA); mAb FK2 anti-Ubiquitin (BML-PW8810-0100; 1:1000; Enzo Life Sciences, Farmingdale, NY); rabbit anti-P62 (1:5000; Dr Gábor Juhász, Eötvös Loránd University, Hungary; Pircs et al., 2012); mAb 6-11B-1 anti-Tubulin (T6793; 1:20,000; Sigma-Aldrich, St. Louis, MO); mAb YL1/2 anti-Tubulin (ab6160; 1:500; Abcam, Cambridge, UK) and rabbit anti-SERCA (1:200; Subhabrata Sanyal, Biogen, Cambridge, MA; Sanyal et al., 2005) antibodies. Actin staining was performed using Alexa-Fluor-568-conjugated phalloidin (A12380; 130 nM; Invitrogen, Carlsbad, CA), and nuclear staining was performed using DAPI (D9542; 300 nM; Sigma-Aldrich, St. Louis, MO).

#### Quantitative analysis of neuronal and glial degeneration

Quantitative analysis of confocal images was performed on maximum projections of image stacks containing a terminal axon branch and an associated glial process (Fig. 3C,D). Terminal axon branches forming synapses were identified using the neuronal plasma membrane marker HRP and the synaptic vesicle marker, Synaptotagmin (SYT). Glial processes were visualized using the glial-specific marker Glutamine Synthetase 2 (GS2). Each axon branch was divided along its long axis into segments of 1 µm in length. As a measure of axonal degeneration, axon fragmentation or ‘beading’ was assessed on the basis of discontinuities in the axon structure. If a segment contained a clearly discernible discontinuity of greater than 0.5 µm in length, the axon structure in that segment was not considered to be continuous. The percentage of segments in which the axon was continuous was taken as a measure of non-degenerated axon structure. The same segments were used for analysis of associated glia, and the percentage of segments in which the glial process was present and continuous was taken as a measure of non-degenerated glial structure. For each condition, analysis was performed on the ten terminal axon branches most centrally located on the medial surface of DLM muscle fiber number 4 in each of three different preparations (*n=*30 terminal axon branches).

#### Mechanical killing of DLM fibers

The DLMs are arranged in two stacks of six muscle fibers, which run longitudinally along each side of the dorsal thoracic midline (Fig. 2A). For mechanical killing of DLM fibers, a tungsten knife was used to penetrate a small region of the cuticle centered over the dorsal surface of the DLM fiber stack to be imaged and was advanced through the entire stack of six DLM fibers. The DLM was not functional after this procedure; however, the flies recovered in general. Death of all muscle fibers was confirmed by their striking opaque appearance, and persistent structural damage was observed by immunocytochemistry. For comparison with preparations exposed to heat shock at 7 days old and processed for immunocytochemistry at 10 days old, mechanical killing was performed at 8 days, corresponding to the end of the HS paradigm, and the preparations were processed at 10 days.

### Electrophysiology

Current clamp methods were used to record the membrane potential of DLM flight muscle fiber numbers 3 and 4. These experiments were performed, essentially, as described previously (Kawasaki and Ordway, 2009). For each condition, recordings were obtained from at least six different cells (n≥6) from four different preparations.

### Western blotting

Western analysis of whole-body fly homogenates was performed using conventional methods, as described previously (Zou et al., 2008). The equivalent of 0.2 flies was loaded per lane. The studies utilized the following primary antibodies: goat anti-HSP70 (sc-27029; 1:250; Santa Cruz Biotechnology, Dallas, TX), rabbit anti-HSP23 (1:100,000; Dr Robert Tanguay, Université Laval, Quebec, Canada), mAb 6-11B-1 anti-Tubulin (T6793; 1:100,000; Sigma Aldrich). Secondary antibodies (LI-COR Biosciences, Lincoln, NE) were generated in donkey: anti-mouse 680LT (925-68022; 1:5000), anti-rabbit 800CW (925-32213; 1:15,000) and anti-goat 800CW (925-32214; 1:5000). Detection was performed using a LI-COR OdysseyCLx imager (LI-COR Biosciences). Western results shown are representative of at least three independent experiments.

### Data analysis

Microsoft (Seattle, WA) Excel was utilized to analyze numerical data and generate graphs. All data values are presented as mean±s.e.m. Statistical significance was determined using the two-tailed Student's *t*-test, and significance was assigned to comparisons for which *P*≤0.05.
